# Large-scale in vitro microdosimetry via live cell microscopy imaging: implications for radiosensitivity and RBE evaluations in alpha-emitter radiopharmaceutical therapy

**DOI:** 10.1186/s12967-023-03991-1

**Published:** 2023-02-24

**Authors:** Remco Bastiaannet, Ioanna Liatsou, Robert F Hobbs, George Sgouros

**Affiliations:** Department of Radiology and Radiological Science, School of Medicine, Johns Hopkins University, 1550 Orleans St, Baltimore, MD 21287 USA

**Keywords:** Alpha emitter, Radiopharmaceutical therapy, Microdosimetry, Single-cell dosimetry, Radiosensitivity, Radiobiology

## Abstract

**Background:**

Alpha-emitter radiopharmaceutical therapy (αRPT) has shown promising outcomes in metastatic disease. However, the short range of the alpha particles necessitates dosimetry on a near-cellular spatial scale. Current knowledge on cellular dosimetry is primarily based on in vitro experiments using cell monolayers. The goal of such experiments is to establish cell sensitivity to absorbed dose (AD). However, AD cannot be measured directly and needs to be modeled. Current models, often idealize cells as spheroids in a regular grid (geometric model), simplify binding kinetics and ignore the stochastic nature of radioactive decay. It is unclear what the impact of such simplifications is, but oversimplification results in inaccurate and non-generalizable results, which hampers the rigorous study of the underlying radiobiology.

**Methods:**

We systematically mapped out 3D cell geometries, clustering behavior, agent binding, internalization, and subcellular trafficking kinetics for a large cohort of live cells under representative experimental conditions using confocal microscopy. This allowed for realistic Monte Carlo-based (micro)dosimetry. Experimentally established surviving fractions of the HER2 + breast cancer cell line treated with a ^212^Pb-labelled anti-HER2 conjugate or external beam radiotherapy, anchored a rigorous statistical approach to cell sensitivity and relative biological effectiveness (RBE) estimation. All outcomes were compared to a reference geometric model, which allowed us to determine which aspects are crucial model components for the proper study of the underlying radiobiology.

**Results:**

In total, 567 cells were measured up to 26 h post-incubation. Realistic cell clustering had a large (2x), and cell geometry a small (16.4% difference) impact on AD, compared to the geometric model. Microdosimetry revealed that more than half of the cells do not receive any dose for most of the tested conditions, greatly impacting cell sensitivity estimates. Including these stochastic effects in the model, resulted in significantly more accurate predictions of surviving fraction and RBE (permutation test; p < .01).

**Conclusions:**

This comprehensive integration of the biological and physical aspects resulted in a more accurate method of cell survival modelling in αRPT experiments. Specifically, including realistic stochastic radiation effects and cell clustering behavior is crucial to obtaining generalizable radiobiological parameters.

**Supplementary Information:**

The online version contains supplementary material available at 10.1186/s12967-023-03991-1.

## Background

The overall survival of patients with early-stage cancer has improved significantly over the past decades, however the prognosis of cancer patients with distant metastases remains abysmal [1, 2]. Radiopharmaceutical therapy with alpha-emitters (αRPT) is increasingly being recognized as a potentially highly effective and safe patient treatment modality for these types of cancers [3, 4].

The highly efficacious nature of alpha radiation can be attributed to its high linear energy transfer (LET), which results in dense and complex DNA damage, resulting in a high probability of cell death [5]. However, the associated short range (50 µm—100 µm, on the order of a few cell diameters) and sparse radiation field at typical activity concentrations greatly complicate accurate dosimetry in patients, as this would require knowledge of the isotope’s distribution at the (sub-)cellular spatial scale. As a consequence, much about the radiobiological response to αRPT remains unknown and current αRPT treatment planning is rarely based on absorbed dose (AD). It is very likely that this results in suboptimal dosing and patient selection [6, 7].

To better characterize the radiobiological response, αRPTs are often studied in vitro using cell monolayers. This allows for the study of agent’s kinetics and cell sensitivity in isolation. Current dosimetric models for these types of experiments commonly idealize cells as spheroids placed in a regular grid (geometric model). A uniform activity which is localized in certain discrete compartments is assumed and AD averages (e.g., s-values) are used for survival modelling.

An example of this is the current MIRD Cell model, in which cells are assumed to be spherical, identical, and are placed in a regular grid [8]. However, it has been suggested in both theoretical [9–14], as well as experimental work [14–17] that cell geometry as well as spatial (intracellular) isotope distribution can have a significant impact on AD for short-ranged particle emissions. Recent efforts have been undertaken to more realistically model groups of cells of different size, geometry, clustering density and associated activity [10]. However, the chosen geometric parameters and assumed cell-specific uptake kinetics of such a model need to sufficiently match the true biological context of the modelled experiments in order to allow generalization of these results (e.g., to in vivo or in patient).

Furthermore, given the stochastic nature of the sparse radiation fields typically associated with αRPTs, the use of microdosimetry is recommended [18, 19]. The statistical variability in cellular AD -which often includes a fraction of cells receiving no AD at all- is thought to be significant at the small spatial scale and associated low levels of activity of individual cells [20, 21]. If this statistical variability is high, then using an average AD or s-value, rather than a microdosimetric approach, would lead an underestimation of cell sensitivity to AD.

To what extent these model aspects are truly necessary for accurate cell sensitivity estimates remains unclear.

The purpose of this study was to systematically measure the individual cell geometry, clustering behavior, and αRPT agent kinetics for a large number of cells under representative experimental conditions, in order to get the most accurate estimates of AD and its microdosimetric variability on a cellular level. Experimental cell survival studies were used to fit cell sensitivity parameters. These data were then compared to simpler geometric models. This allowed for the assessment of which model aspects are crucial for accurate radiobiological modelling of cell survival assays after treatment with αRPT.

## Methods

The specific AD distributions and alpha particle track lengths through the nucleus were calculated for real cell geometries and antibody (Ab) kinetics under experimental conditions. The cell geometries and Ab kinetics were measured using high-resolution dynamic 3D confocal microscopy. For each time frame, the full physics of the isotope decay was simulated in a Monte Carlo framework. To this end, the nuclei were segmented and imported into a custom-build GEANT4 program, together with the measured Ab spatial distribution for each time frame. Simulated events were saved individually, which enabled efficient microdosimetry calculations for each cell, source location, time frame and Ab concentration. These results, together with previously published cell survival data, were used to model the likelihood of survival on a single-cell level. The results for this detailed model were compared to the results of simpler geometric models.

### Data acquisition

#### Cell line and fluorescent staining

The experimental conditions of a previous in vitro cell survival experiment were replicated for this study [22]. The cell line which was used, is a syngeneic murine rat HER2/neu expressing cell line which is a mouse cell line that expresses rat HER2/neu receptors [23, 24]. The cells were cultured in the appropriate media, but the base media was changed to a non-phenol red RPMI1640. The cells were seeded in thin bottomed well plates, optimized for high-resolution imaging (Ibidi µ-Slide 2 well) the day before imaging experiments, to allow for adherence.

A relevant HER2/neu monoclonal antibody, 7.16.4 (BioXCell) was labelled with the Alexa Fluor 488 labelling kit (Thermo Fisher Scientific). Cell nuclei were counter stained with Hoechst 33342 20 min before imaging started. A separate imaging batch of cell culture media was stained with biologically inert dextran (10,000 MW, neutral; Thermo Fisher Scientific), labelled with Texas Red at a concentration of 2.5 µM. This media (“negative”) stain provided an outline of the individual cell geometries and was added to the incubation wells only moments before imaging started.

### Data scans

Imaging was performed on live cells with a Zeiss LSM 880 using a 63 × PlanApo oil emersed objective. The voxel size was 0.101 × 0.101 µm in-plane and 0.159 µm in the vertical direction. Laser power and filter settings were optimized once on a test batch of cells and kept constant for all subsequent experiments. The environmental chamber was kept at 37 °C with 5% CO_2_.

Time lapse acquisitions at 12 frames per hour were made for antibody incubation times for up to 120 min. All other acquisitions were static.

### Photobleaching correction

Photobleaching in time lapse acquisitions was modelled with a normalized double-exponential as a function of the number of laser passes through a voxel. The parameters were found by fitting the double-exponential to the decaying signal of formalin-fixed (4%) cells which had been incubated in 10 nM labelled antibodies for at least 2 h and imaged repeatedly. All measured antibody signal time lapses were corrected for photobleaching using this model.

### Cell and nucleus segmentation

Individual cells were segmented based on the stained media signal in which cell boundaries were clearly visible. In short, the segmentation pipeline consisted of adaptive histogram equalization and Laplacian edge enhancement, followed by a water shedding segmentation algorithm. More details on the segmentation processing steps may be found in the Additional file 1.

Nuclei were segmented by a chain of preprocessing steps (see Additional file 1), followed by a morphological Chan & Vese segmentation [25].

The cell segmentations were shrunk 0.2-0.3 µm by binary erosion to obtain a segmentation of the cells’ cytosol. The cell segmentations were then expanded by use of a distance transform. Subtracting the cytosol from the expanded segmentation resulted in the segmentation of the cell membrane compartment.

### Time frame coregistration

Live cell motility resulted in some cells moving in and out of the field of view during time lapse imaging. To enable consistent identification of individual cells, the different time frames were coregistered to a single reference frame before segmentation using a group-wise registration implemented in SimpleElastix [26]. The nuclei were segmented as described above. These nucleus segmentations were subsequently inverse transformed to their original positions and cell segmentation was continued as described above.

### Correcting for selection bias

Manually selecting a section of the well to image may lead to selection bias. An unbiased cell density distribution was therefore measured and subsequently used to correct any collected data.

Cell density patterns were measured by making overview scans of the Hoechst channel of large parts of the well. Constant imaging depth relative to the well bottom was maintained using the Definite Focus II module.

The images were contrast enhanced (CLAHE) and segmented using a Otsu threshold [27]. The resulting binary mask was separated into individual nuclei using water shedding, followed by a connected component analysis (details in Additional file 1). The center of mass (CoM) of each nucleus mask was used to calculate the Euclidian distance between every pair of nuclei. From this, the unbiased cell density distribution was constructed.

The cell density distribution was measured in the 3D dynamic data set as well and a weight was assigned to each cell such that the reweighted cell density distribution closely resembled the unbiased cell density distribution. These weights were found using a Powell optimizer and were used in all subsequent cross-dose and radiobiological calculations.

### Pharmaco-kinetic modelling of antibody kinetics

A pharmaco-kinetic (PK) model was fit to the measured number of antibodies in the membrane and cytosol compartments for all cells together as a group (i.e., ensemble fit). This made it possible to estimate antibody kinetic parameters at any time point and at any Ab concentration.

 Calibration was required to translate microscope image intensity to number of antibodies. To this end, a binding assay was performed at 37 °C and 5% CO_2_, replicating experimental conditions, which allowed for natural processes like internalization to occur. The 7.16.4 antibody was labelled with ^111^In using the DTPA chelator, and immunoreactive fraction (IRF; fraction of Ab that is able to successfully bind with its antigen after labelling) and specific activity were measured, as described previously [22]. A known number of cells were seeded in 6-well plates and allowed to adhere overnight. The cells were then incubated at 37 °C for either 4 h with a varying molar Ab concentration (0.25–30 nM) or with a set Ab concentration (10 nM) for a varying duration (5 min to 24 h). All conditions were executed in triplicates. After incubation, cells were thrice washed with PBS and the entire cell fraction was dissolved in 5% SDS and activity was counted in a gamma well counter. Normal cell growth was assumed, with a cell doubling time of 26 h and the average activity per cell was calculated. The average activity per cell was converted to number of cell-associated antibodies per cell, by using the specific activity. Differences in binding efficacy were corrected for using the ratio of IRFs to translate the found binding affinity for ^111^In-labelled Ab to ^212^Pb-labelled Ab [28]. This, together with the specific activity of ^212^Pb-labelled Ab was used to calibrate the microscope images and convert to ^212^Pb activity per voxel [22].

**Fig. 1:**
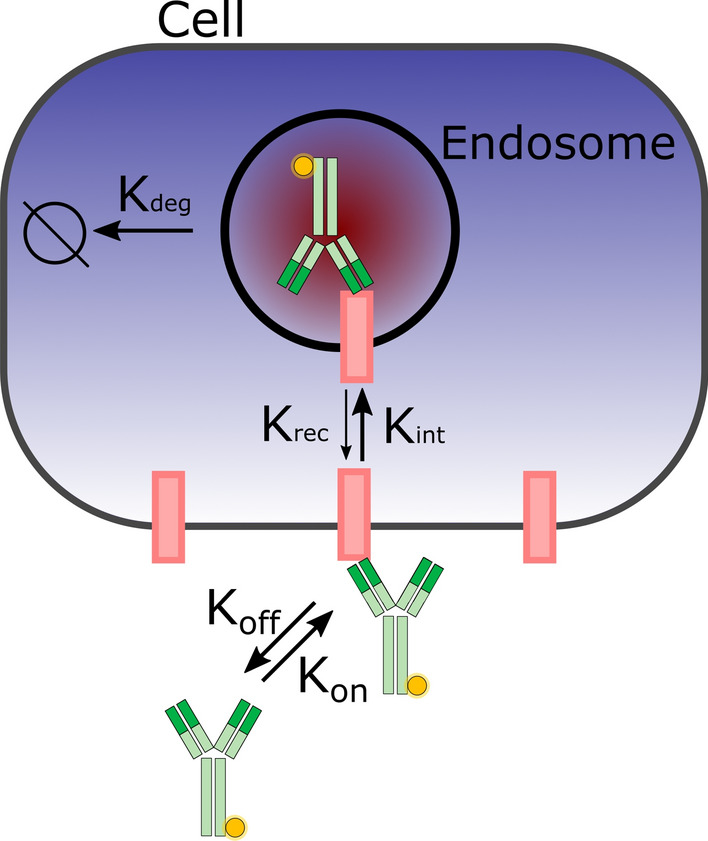
Design of the pharmaco-kinetic model of antibody kinetics. This model describes antibody-receptor association rate (K_on_) and dissociation rate (K_off_), antibody-receptor complex internalization rate (K_int_), antibody degradation rate (K_deg_), and receptor recycling rate (K_rec_)

The PK model shown in Fig. 1 was fit to the measured number of antibodies in the cytosol and membrane compartments using a Nelder-Mead Simplex optimizer. The objective function was the L2-norm of the sum of square differences between the predicted and measured number of antibodies in each compartment. For each measured time point, 95%-confidence intervals were calculated.

### Monte Carlo physics simulation

Radioactive decay and particle transport through matter was simulated using GEANT4 (v 10.06 patch 2) [29]. All matter was set to be equivalent to water. The cell nucleus was assumed to be the relevant dose receiving target for cell survival. We define a primary event as the decay of a single ^212^Pb atom, including the decay of all its daughters to stable ^208^Pb. All decays are assumed to occur in situ.

The segmented nucleus masks were transformed to VTK mesh models by extracting the iso-surface of the masks, followed by smoothing, cleaning, and reducing operations. These mesh models were subsequently saved to a non-binary STL file format, which could be read into the custom-build GEANT4 program using CADMesh [30].

The spatial antibody signal from the confocal images was used as the probability distribution for the random selection of primary event locations. This ensured that the overall distribution of primary events followed the measured spatial distribution of the antibodies exactly.

To this end, the spatial antibody signal from the confocal images was saved in the NRRD file format, which retains the spatial coordinates of each voxel. These files were then imported into a custom-build GEANT4 program.

Primary events were simulated for each target nucleus individually and were stratified according to AD originating from the (i) cell membrane (membrane AD); (ii) cell cytosol (cytosol AD); (iii) cell neighborhood (cross AD). This was done by masking out all antibody signal which was not part of the compartment of interest. Then, 1 million primary events, which included all daughters and secondary particles, were simulated (10 million for cross AD). The track lengths of alpha particles passing through the target nucleus and the deposited energy were saved separately for each individual primary event.

Some cells were close to the edges of the field-of-view, which would bias the estimation of cross AD for these cells. Therefore, activity in the surrounding area was approximated by extending the antibody signal with copies of the signal in both directions, creating a 3 by 3 tile.

### Monte Carlo-based microdosimetry

Microdosimetry describes the dosimetric implications of the stochastic nature of low fluence radiation, which is relevant when studying alpha emitters, especially on the small spatial scale of single cells and the associated low number of decays. In the microdosimetric literature, rather than using a single value for deposited energy or AD, the deposited energy is a stochastic variable called specific energy $z$, which has a probability distribution $f\left(z\right)$. These specific-energy distributions can be derived analytically for simple, well-defined geometries and uniform source locations [20].

In more complex situation, like in this study, specific-energy distributions may be estimated using repeated Monte Carlo simulations. In short, a number of $n$ primary events are simulated and the total specific energy for each primary event is scored. The number, $n$, is a Poisson-distributed stochastic variable for which the mean, $\langle n\rangle$, corresponds to the expected total number of primary events in a source region. This is then repeated many times (e.g., 1 million times). The resulting distribution of the scored quantities of interest approaches the true specific-energy distribution for larger numbers of repetitions.

However, performing these simulations for each experimental condition (e.g., concentration) and each time frame (to account for Ab kinetics) would be prohibitively computationally demanding. To maintain feasibility, a large number of primary events (1 million; 10 million for cross dose) were simulated in one session and for each primary event, the total deposited energy and track length through the nucleus was scored. We then used a bootstrapping approach to estimate the specific energy and track length distributions for each experimental condition. This allowed for efficient reuse of previously calculated primary events, decreasing the computational burden significantly. Each microdosimetric distribution presented in this manuscript is based on 1 million bootstrap folds.

Because the Ab kinetics were included in this model, the number of primary events was calculated in small time steps. The expected value for the number of primary events per time step was used as input for the bootstrapping method for each individual cell and compartment.

### Radiobiological modelling

Cell surviving fraction (SF) as a function of activity concentration, normalized to a sham-treated 0 Gy group, was measured previously [22]. We used the microdosimetric methods, as well as the cell density reweighting approach as described above, to estimate the unbiased AD and track length distribution for every experimental condition of previously reported SF data.

Specific energy distributions for each cell, $f\left(z\right)$, were scored in a histogram with $K = 250$ equal bins, indexed with $i$. The likelihood of an individual cell surviving, $P\left(S\right)$, was modelled as:

1 $$\mathrm{P}\left(\mathrm{S}\right)=\sum_{i=1}^{K}{{w}_{i} e}^{-{z}_{0}{z}_{i}}$$

where z_0_ is an intrinsic microdosimetric cell sensitivity parameter and ${w}_{i}$ is defined as:

2 $$w_{i} = {\raise0.7ex\hbox{${z_{i} }$} \!\mathord{\left/ {\vphantom {{z_{i} } {\mathop \sum \nolimits_{i = 1}^{K} f(z_{i} )}}}\right.\kern-0pt} \!\lower0.7ex\hbox{${\mathop \sum \nolimits_{i = 1}^{K} f(z_{i} )}$}}$$

Then, the survival fraction of a colony is the average of the survival probabilities of all the cells in that colony. We considered our data set to be a biased sample of a much larger hypothetical colony. To correct for this selection bias, a weighted average (rather than a simple average) was taken of the individual cell survival probabilities. As such, the expected value for survival fraction, $\langle SF\rangle$, for a number of $J$ cells is given by:

3 $$\left\langle {SF} \right\rangle = \mathop \sum \limits_{j = 1}^{J} {\raise0.7ex\hbox{${c_{j} P\left( {S_{j} } \right)}$} \!\mathord{\left/ {\vphantom {{c_{j} P\left( {S_{j} } \right)} {\mathop \sum \nolimits_{j = 1}^{J} c_{j} }}}\right.\kern-0pt} \!\lower0.7ex\hbox{${\mathop \sum \nolimits_{j = 1}^{J} c_{j} }$}}$$

where ${c}_{j}$ indicates the bias correction weights.

This was compared to the classical approach, where cell sensitivity $\kappa$ is estimated by fitting a decaying mono-exponential to the average nuclear AD across all cells, ignoring radiation stochastic effects:

4 $$\mathrm{SF}={e}^{-\upkappa \overline{\mathrm{D}} },$$

with $\overline{D }$ the selection-bias-free average AD in Gray and $\kappa$ the radiosensitivity parameter in Gy^−1^.  $\overline{D }$ was calculated as:

5 $$\overline{D} = \mathop \sum \limits_{j = 1}^{J} {\raise0.7ex\hbox{${c_{j} \left\langle D \right\rangle_{j} }$} \!\mathord{\left/ {\vphantom {{c_{j} \left\langle {D} \right\rangle } {\mathop \sum \nolimits_{j = 1}^{J} c_{j} }}}\right.\kern-0pt} \!\lower0.7ex\hbox{${\mathop \sum \nolimits_{j = 1}^{J} c_{j} }$}}$$

where$\left\langle D \right\rangle_{j}$ indicates the expected value for AD in cell nucleus $j$, which was calculated for cell $j$ as:

6 $$\left\langle D \right\rangle = \frac{{\mathop \sum \limits_{i = 1}^{K} f\left( {z_{i} } \right) \cdot z_{i} }}{{\mathop \sum \limits_{i = 1}^{K} f\left( {z_{i} } \right)}}$$

Radiosensitivity parameters ${z}_{0}$ and $\kappa$ were found by minimizing the sum of squared errors between the predicted SF and the experimentally measured SF using a Nelder-Mead simplex optimizer.

### Radiosensitivity parameter uncertainty

Non-parametric 95%-confidence intervals (95%-CI) of the radiobiological parameters were estimated using a bootstrapping procedure where possible. In each iteration, a subset of the data was selected to which the radiobiological parameters were fit. This was repeated 500 times, resulting in a sample distribution of the fitted parameters. The range between the 2.5 and 97.5-percentile of this distribution is the two-sided 95%-CI and was used for statistical significance testing between radiobiological models.

### Reference geometric model

The cells in a reference geometric model were modelled as two concentric spheres where the inner sphere represented the nucleus and the outer sphere the cytosol and cell membrane. These cells were placed in a regular hexagonal grid, with the cell spacing chosen such that the cells were maximally spaced out over the well plate bottom. The diameters of the entire cell and nucleus were set to 18 µm and 10 µm, respectively.

## Results

A total of 567 unique cells were included for analysis. Multiple time frames between 10 min and 26 h post incubation were acquired. Antibody kinetics between 0- and 120-min post incubation were recorded using time lapse acquisitions, whereas the time between 120 min and 26 h post incubation were sampled using static snapshots. Correction for photobleaching was included. Details can be found in the Additional file 1.

**Fig. 2:**
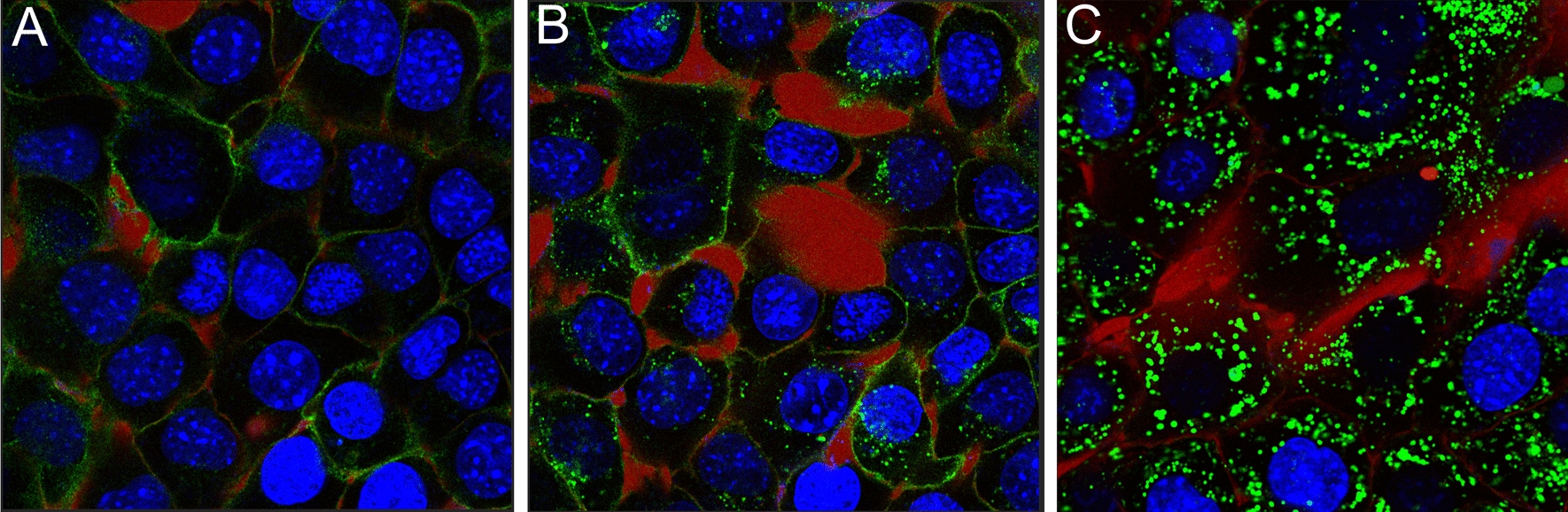
Evolution of antibody kinetics over time. The Initial membrane binding, shown at 10 min post incubation (**A**), internalization (shown at 100 min post incubation) in (**B**) and finally, pooling of the antibody-rich endosomes, likely into lysosomes (26 h post incubation) in (**C**). Stained media is in red, antibodies green and the nuclei are blue. Timelapse video can be found under additional materials (additional file 2)

For the purpose of this study, we have identified three distinct phases in antibody binding and trafficking, which are shown in Fig. 2: (i) membrane binding; (ii) internalization; (iii) pooling into endosomes.

### Segmentations

**Fig. 3:**
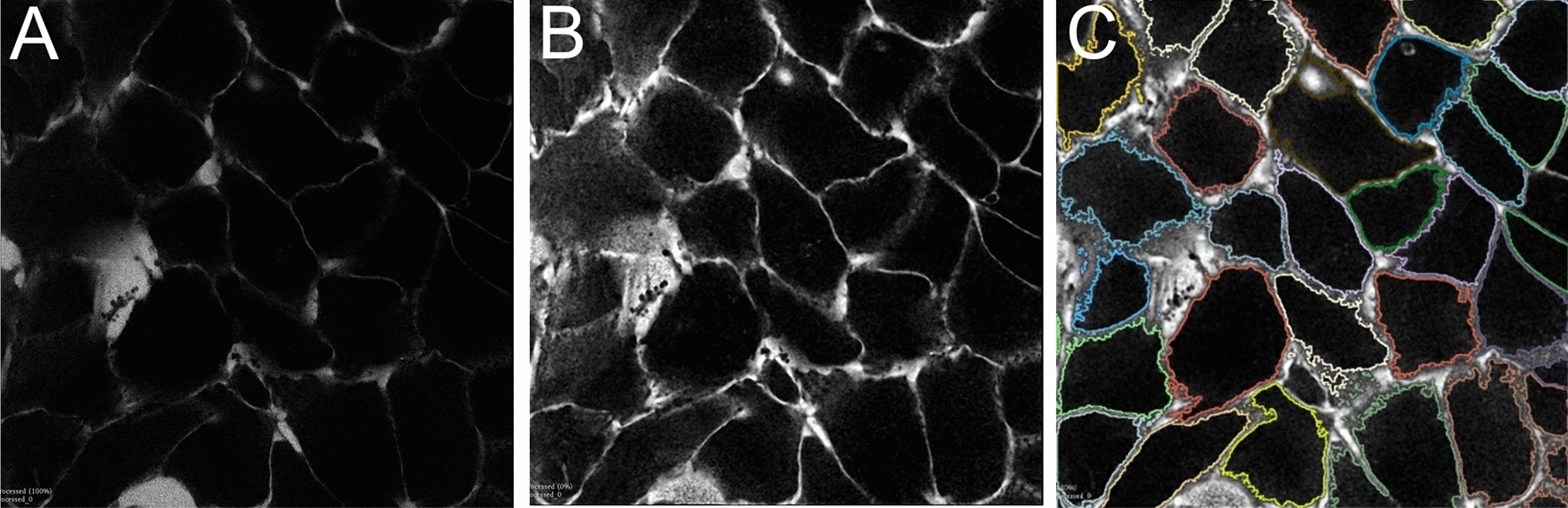
Segmentation of the cell contours. Original stained media signal in (**A**), contrast enhanced in (**B**) and final segmentation in (**C**). These final masks where then eroded by 0.2 µm, which resulted in the mask for the cytosol

**Fig. 4:**
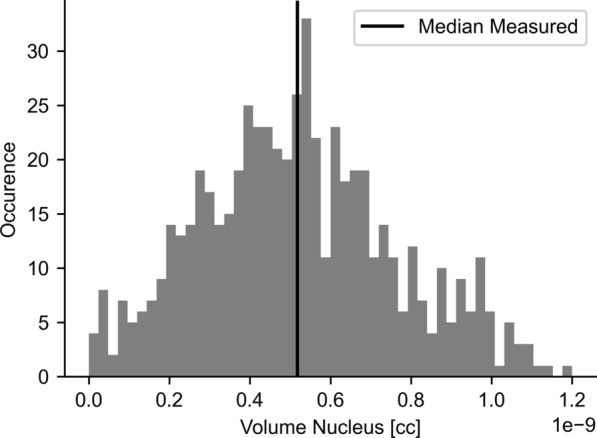
Histogram of nucleus volumes. Vertical black bar indicates median volume, which corresponds to a sphere-equivalent nucleus diameter of 10 µm. Extreme low and high volumes are a result of over and under segmentation, respectively, by the segmentation algorithm

All segmentations were visually inspected. Typical cell segmentation results are shown in Fig. 3. A histogram of measured nuclear volumes is given in Fig. 4. The median nuclear volume corresponded to an equivalent sphere with a 10 µm diameter.

### Acquisition bias

**Fig. 5:**
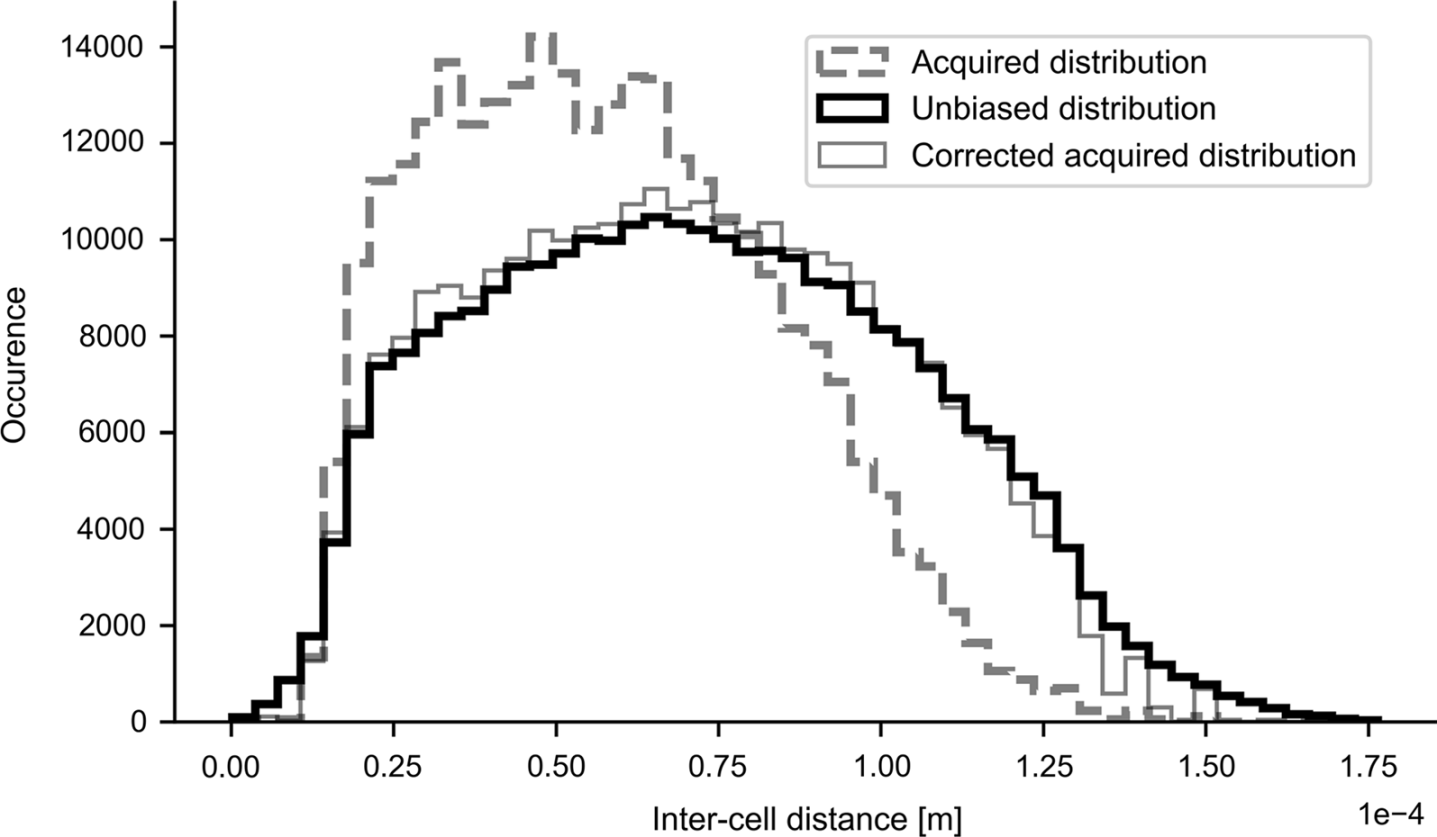
Original nucleus density histogram, extracted from high-resolution scans (Acquired distribution) and the unbiased density distribution as measured in the unbiased tile scans. The acquired data was reweighted such that it matched the unbiased distribution. This reweighted distribution was used for all subsequent AD and cell survival calculations

Automated overview scans of a 10.9 mm^2^ surface area, containing a total of 1.2 × 10^4^ cells were acquired. Cell nuclei were automatically detected in these scans and the distribution of inter-cell distances was estimated for each field-of-view (*Unbiased distribution*). The distribution of inter-cell distances for each high-resolution data scan was also estimated and was found to be skewed, relative to the unbiased overview scans (*Acquired distribution*), indicating a clear acquisition bias (Fig. 5). Each cell’s data was reweighted such that the overall inter-cell distance distribution closely matched the unbiased distribution. These weights were used for subsequent AD and cell survival fraction calculations.

### Antibody kinetics

**Fig. 6:**
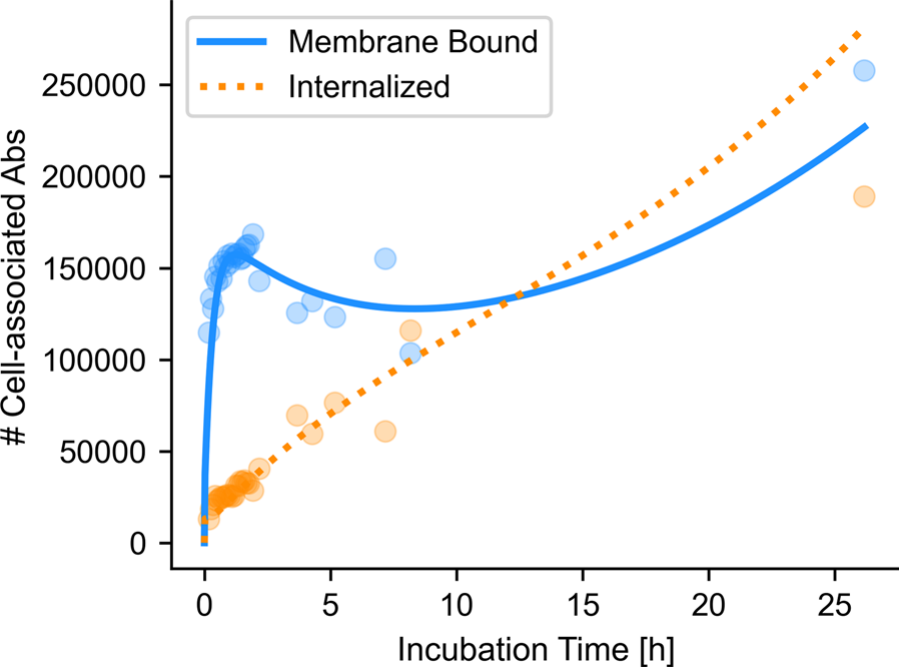
PK fit to measured number of antibodies per cell over time. Each dot indicates the average of the cells in a single microscope field-of-view

The number of associated antibodies with the cell membrane and cytosol was modelled using the PK model design of Fig. 1. The resulting binding and internalization time curves are shown in Fig. 6 for the measured 10 nM Ab concentration. The optimization resulted in an average of 1.72 × 10^5^ effective binding sites per cell and an equilibrium dissociation constant, K_D_, of 0.98 nM for the ^111^In-bound Abs, which corrected for difference in immunoreaction fraction (95% for ^111^In; 55% for ^212^Pb), corresponds to 1.69 nM for ^212^Pb-bound Abs.

### Mean absorbed dose trends

**Fig. 7:**
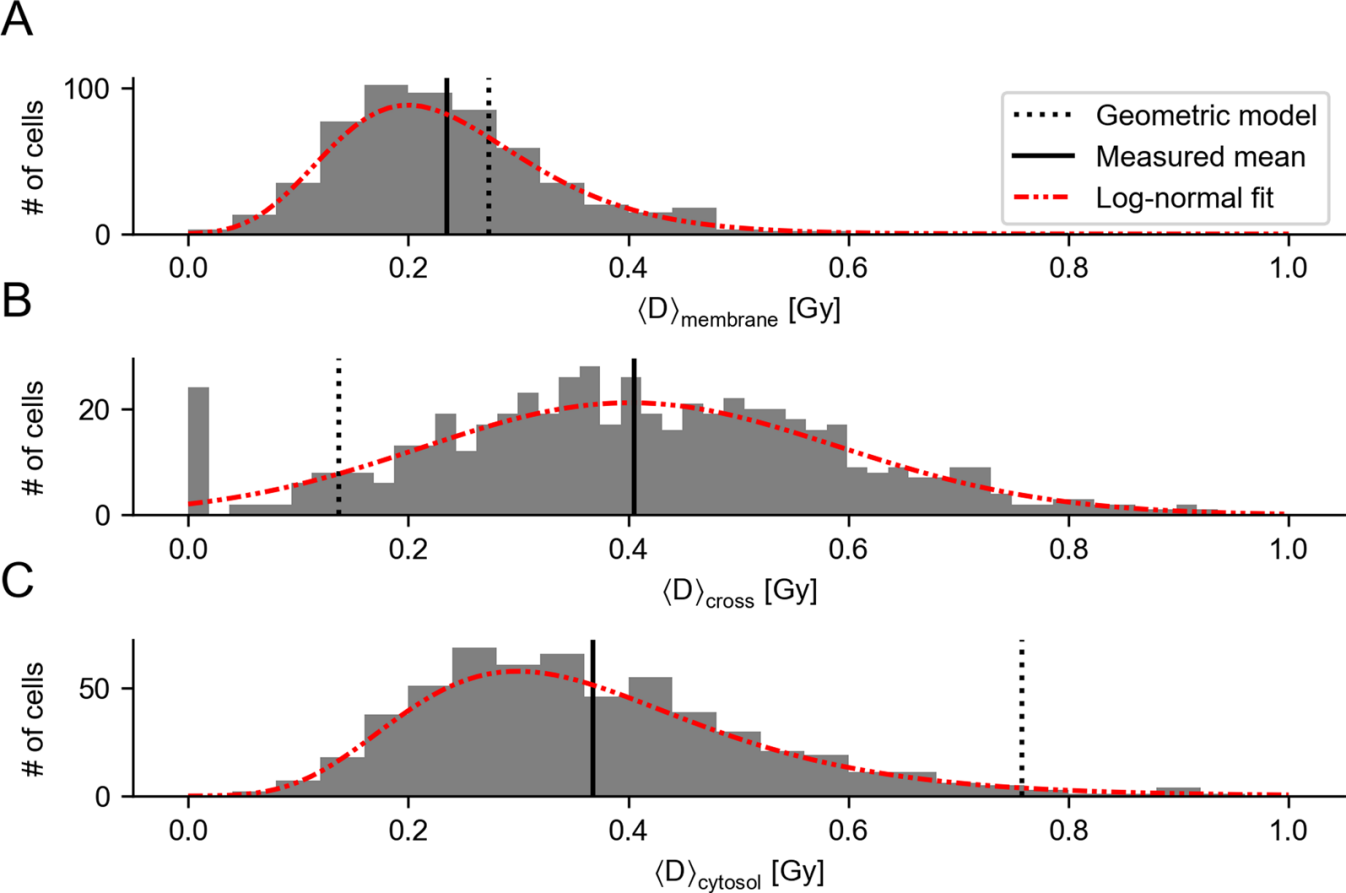
Histograms of mean ADs for individual cells at the highest activity concentration. **A** Mean AD of energy emitted by membrane-bound activity. **B** Mean AD for radiation received from neighboring cells. **C** Mean AD imparted on the nucleus by internalized activity. All ADs are log-normally distributed over cells. Vertical dashed bar indicates the reference value of the geometric model; the solid black line indicates average of single cell calculated values

In order to assess the inter-cell variability of ADs, the distribution of mean AD, $\langle D\rangle$, over the different cells is shown in Fig. 7 for the highest activity concentration. Membrane, cytosol and cross mean ADs are all log-normally distributed (Kolmogorov–Smirnov p = 0.61, 0.96 and 0.84 respectively).

The average of the estimated mean ADs for membrane-to-nucleus was relatively close to what was calculated using the geometric model (16.4% difference). Conversely, the geometric model estimate for cross cell AD was larger (factor 2 difference), illustrating the difference in cell clustering behavior between the two models.

**Fig. 8:**
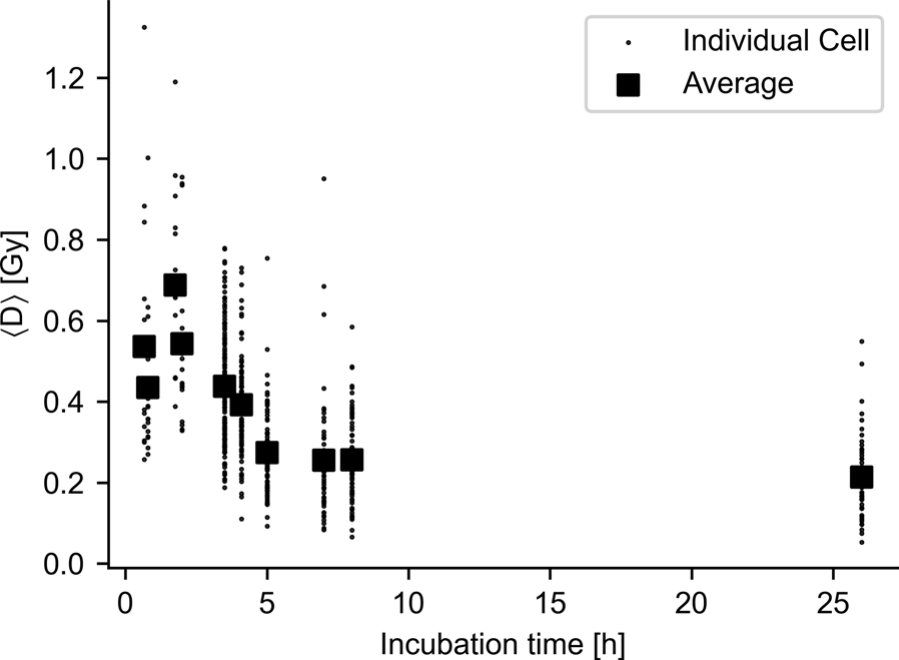
Changing cytosol average AD over time. The temporal decrease shown here, visually coincided with the occurrence of endosomal pooling. For visual clarity, time frames were grouped by acquisition session

The estimated cytosol mean ADs had a relatively wide dispersion, implying varying geometric patterns. This is corroborated in Fig. 8, where the cytosol mean ADs are plotted as a function of time, revealing a decrease over time. This process visually coincided with endosomal pooling, an example of which is shown in Fig. 2C.

### Microdosimetric distributions

**Fig. 9:**
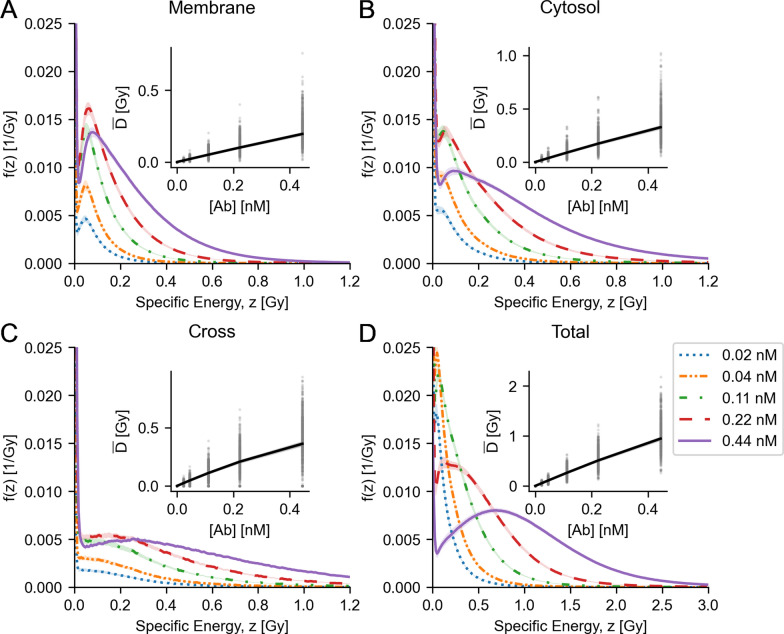
Microdosimetric specific energy distributions, averaged over cells, for nuclear AD by membrane-bound activity (**A**), cytosolic activity (**B**), cross activity (**C**) and the total activity (**D**). Inserts show the expected value for ADs, averaged over cells as a function of Ab concentration. Dots indicate expected values for individual cells. Shaded areas in the inserts indicate 95th-percentile. Shaded areas elsewhere indicate bootstrapped 95% confidence intervals for the means

**Fig. 10:**
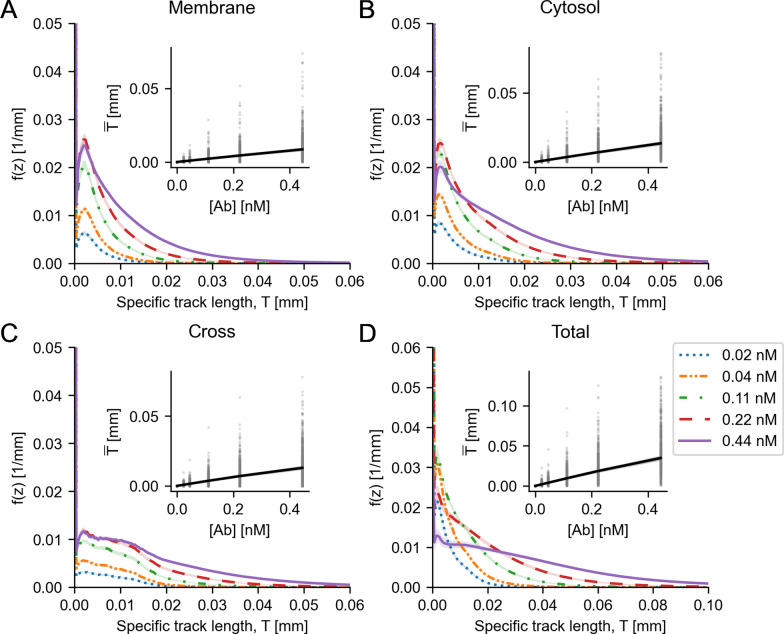
Microdosimetry average track length frequency distributions for **A** membrane AD, **B** cytosol AD, **C** cross AD and **D** total AD. Inserts show the expected values for the track lengths, averaged over all cells. Dots indicate expected values for individual cells. Shaded areas in the inserts indicate 95th-percentile. Shaded areas elsewhere indicate bootstrapped 95% confidence intervals for the means

**Fig. 11:**
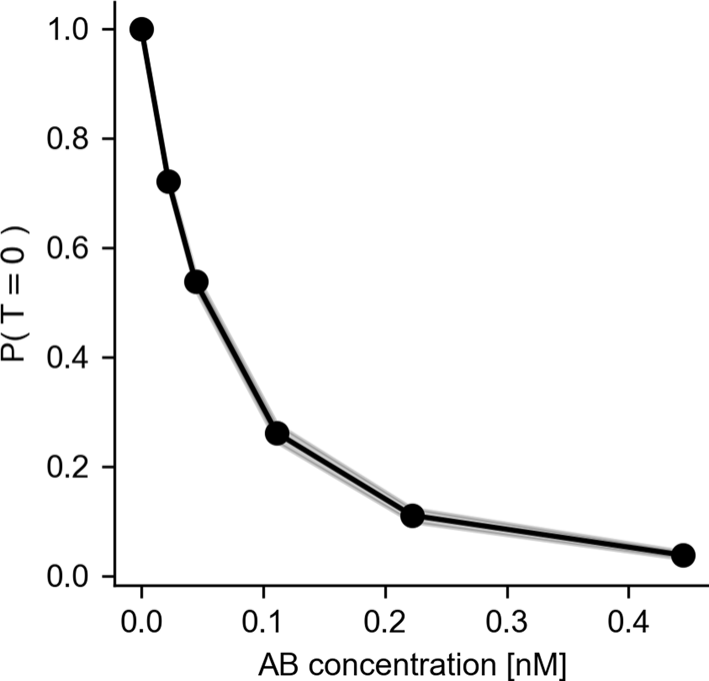
Zero-hit likelihood over all cells. Lower half of the modelled Ab concentrations resulted in more than 50% of the cells receiving no dose at all. Shaded area indicates bootstrapped 95% confidence interval for the mean

**Fig. 12:**
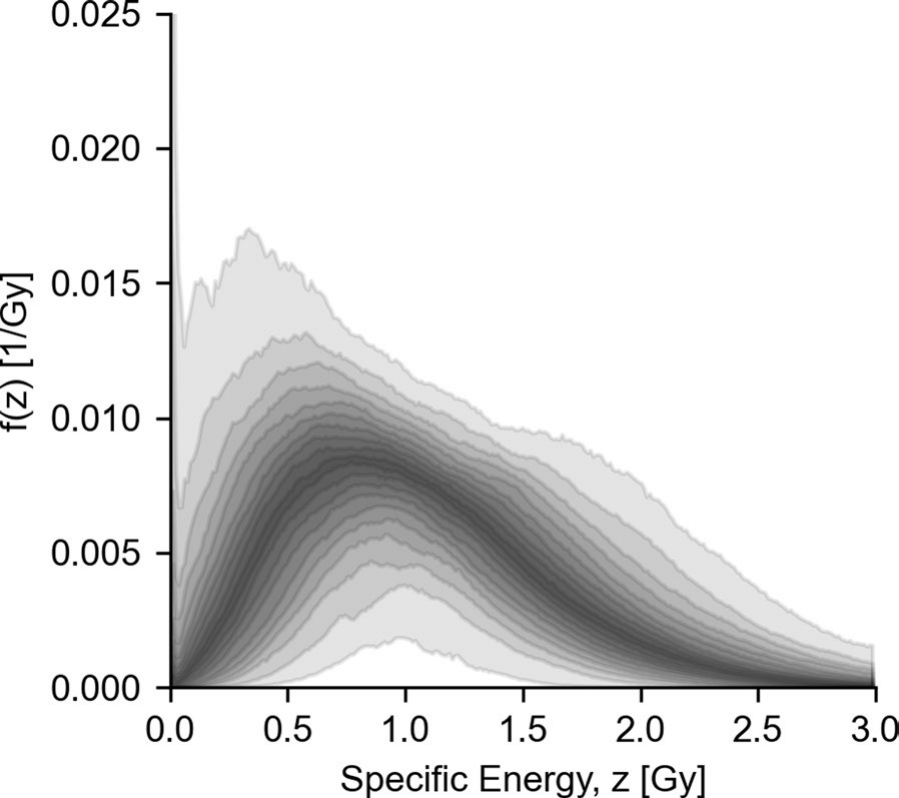
Density plot indicating dispersion around the mean for total absorbed specific energy at the highest concentration for individual cells (compare to Fig. 9D). Plot is color coded, so that the 95-percentile is denoted with the lightest gray and the 5-percentile with the darkest gray

Microdosimetric probability distributions were calculated for cell nucleus AD (specific energy distribution; Fig. 9) and alpha particle track length (specific track length distribution; Fig. 10). Of note is the relatively wide distribution for both AD, as well as track length. The specific energy distribution for the total of all compartments (Fig. 9D) shows a peak AD to and including 0 Gy, especially for lower Ab concentrations. This indicates the likelihood of a cell not being hit by any radiation (zero path length trough the nucleus), also known as the zero-hit likelihood. A sharp decline of the zero-hit likelihood was observed as a function of Ab concentration, shown explicitly in Fig. 11. The variability of single-cell level specific energy distributions is shown in Fig. 12.

**Fig. 13:**
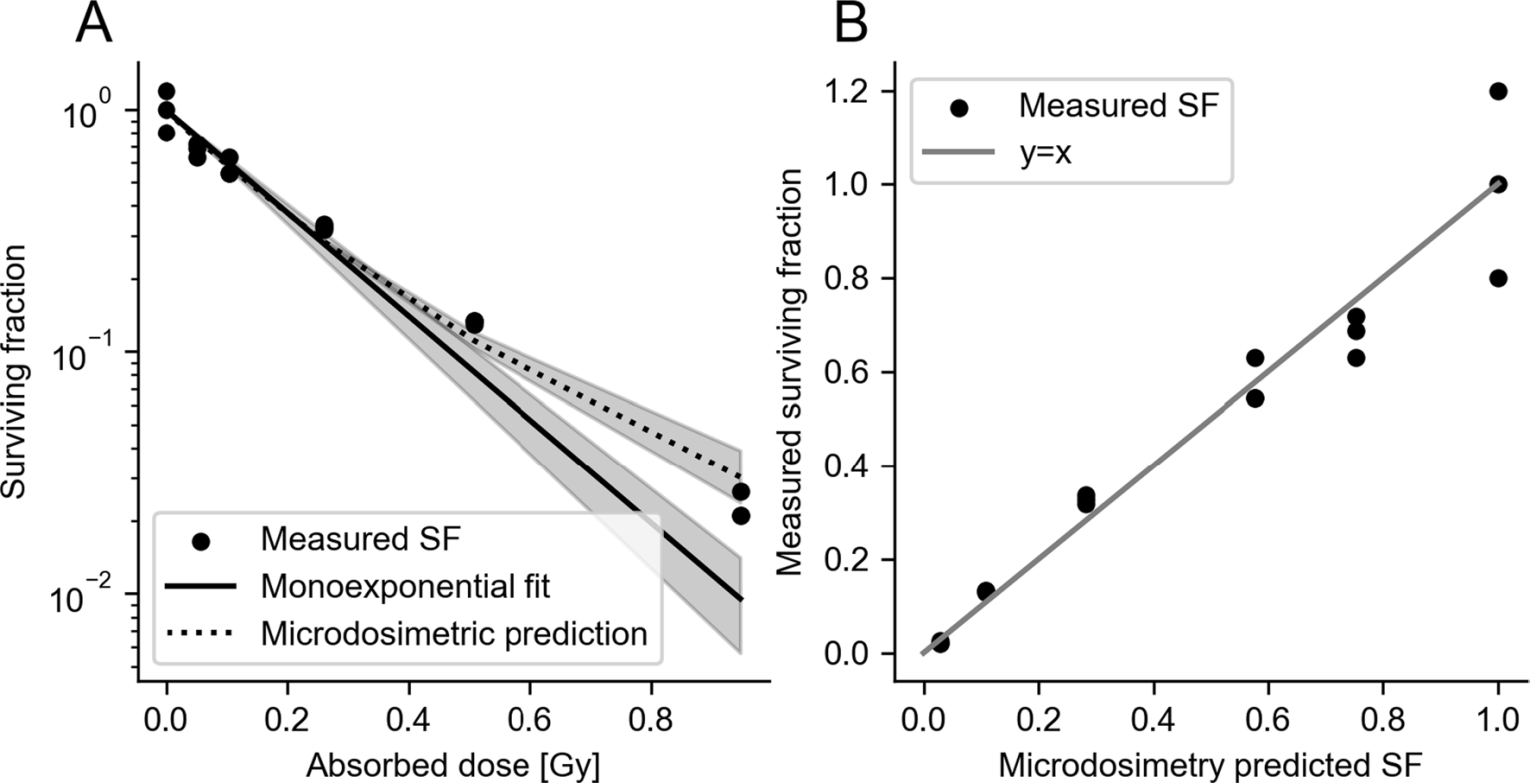
Microdosimetric SF modelling. **A** SF as measured and modeled with a monoexponential function with AD and the microdosimetric approach. The microdosimetric approach results in more accurate predictions for SF than the monoexponential fit. Shaded areas indicate bootstrapped 95% confidence intervals. **B** microdosimetry SF prediction correlates well with measured SF

**Fig. 14:**
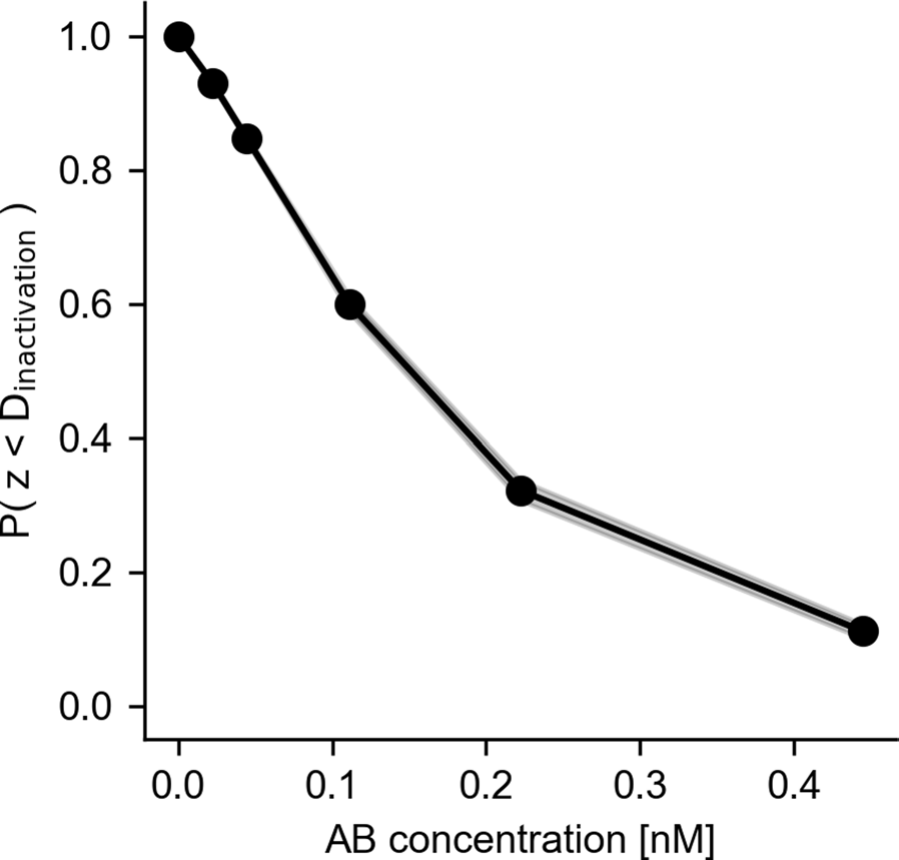
Likelihood of an AD which is lower than the mean inactivation dose, ${D}_{inactivation}$, over all cells. Shaded area indicates bootstrapped 95% confidence interval for the mean

From these specific distributions, the expected value for AD, averaged over cells, was calculated for every Ab concentration. The SF was plotted against these averaged ADs (Fig. 13A). The mean inactivation dose, ${\overline{D} }_{inactivation}$, which is the area under this curve, was calculated using the trapezoid integration method. ${\overline{D} }_{inactivation}$ was 0.24 Gy. The average likelihood of a cell absorbing less than the mean inactivation dose as a function of Ab concentration was calculated and plotted in Fig. 14.

### Radiobiological parameters

The previously measured SFs were used to estimate cell sensitivity parameters [22]. The cell sensitivity to mean AD, $\kappa$, and the microdosimetric cell sensitivity parameter, ${z}_{0}$, were found by optimizing the sum of squared error between the measured and predicted SF for each AD using the Mead-Nelder Simplex optimizer. This resulted in a $\kappa$ of 5.0 Gy^−1^ (95%-CI 4.4–5.6) and a ${z}_{0}$ of 30.1 Gy^−1^ (95%-CI 18.5–64.3). The predicted SFs for each approach are shown in Fig. 13A. The microdosimetric approach predicted SF significantly better than the average dose approach (sum of squared error 0.00797; 95%-CI 0.0065–0.00950 versus 0.0155; 95%-CI 0.0145–0.0191), especially for the higher doses. A direct correlation between predicted and measured SF is shown in Fig. 13B. This is due to the non-linear behavior of microdosimetric quantities with increasing Ab concentration, as is also evidenced by the non-linearities in Fig. 11.

### Relative biological effectiveness

**Fig. 15:**
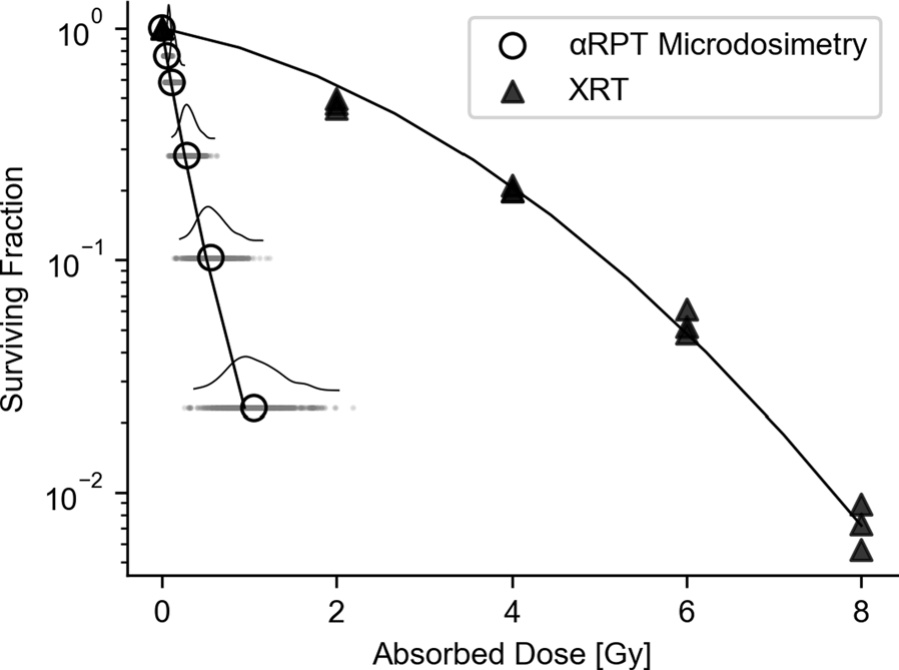
Graphical overview of mean AD estimates for each cell for αRPT and EBRT in one plot. Each dot indicates the mean AD, $\langle D\rangle$, of each measured cell. The curve above indicates the relative number of cells at that position. The ratio between the XRT and αRPT lines for a given SF is the RBE metric

RBE values were calculated from the SF fits for both αRPT and previously published external beam radiotherapy (EBRT) data [22, 31]. AD-survival data for EBRT was fit with a linear-quadratic equation (α: 0.169; β: 0.056). The resulting RBE values for the geometric model, microscopy-based non-stochastic model and microdosimetric $z$ are reported in Table 1. The RBE estimates are significantly different between the Geometric Model and the non-stochastic microscopy-based model. However, adding microdosimetry to the latter does not result in significantly different RBE estimates. An overview of the AD data is graphically represented in Fig. 15.

**Table 1:** Calculated RBE values. Subscripts indicate corresponding SFs; RBE2 denotes a SF scale-free RBE metric

RBE Metric	Geometric Model	Non-stochastic AD (95%-CI)	Microdosimetric AD
RBE_10_	8.5	10.8 (9.9–12.0)	9.0
RBE_37_	11.5	14.7 (13.4–16.3)	14.6
RBE_50_	12.9	16.5 (15.1–18.3)	17.5
RBE_2_	13.7	17.5 (16.0–19.4)	–

## Discussion

In this study, we have systematically measured both cell geometries as well as the relevant biological processes in situ of a large number of cells under typical experimental conditions. We used this to establish realistic estimates of AD, its variability, microdosimetry and studied the implications of several common model selections on cell sensitivity and RBE estimates.

The largest discrepancy between the reference geometric model and our direct estimates are in the estimates for cross AD (Fig. 7B). Cross AD is mostly affected by cell-to-cell proximity, which in this study was measured directly and was corrected for acquisition bias (Fig. 5). The uniform cell clustering density of the geometric model corresponds to larger inter-cell distances than those found under typical experiment conditions where cells tend to tightly cluster together (e.g., Fig. 2). Cell clustering results in a higher cross AD (Fig. 7B). This highlights the fact that careful consideration of cell-to-cell proximity and cell clustering behavior is crucial for any AD model.

Conversely, we found that, on average, the impact of cellular geometry on membrane-originating AD is limited when cells are cultured in groups, as evidenced by the small difference between the mean AD estimate of our model and the geometric model for the membrane (Fig. 7A). This suggests that, although there is variability due to cell geometry, on average, when cells become adherent, but are closely packed, the both nucleus and cell membrane change their shapes in such a way that any geometric effect of cell shape change on membrane-originating AD is limited and is probably counterbalanced by the location and flattening of the nucleus. Our results contrast with the findings by Guerra Liberal, et al., who found that the AD was significantly different for an attached (i.e. flattened) and a concentric (i.e. spherical) model [10]. One reason for this discrepancy with our findings could be that in their study, the location and the shape of the nucleus was modelled instead of directly measured. Tang, et al., modelling electron sources, have found a major impact of cell geometry on nucleus AD, using two measured 3D models of a cell membrane and nucleus [11]. Similarly, Arnaud, et al. found that for Auger emitters, realistic cell models are needed, especially when a non-uniform source distribution is expected [9]. Although their results are internally valid, the models in both cases were based on single cells in isolation. As such, these cell geometries might be less representative of cells under typical experimental conditions, which are packed into clusters, as described above.

The NT2.5 cells used in this study readily internalized membrane bound antibodies into endosomes (Fig. 2B, C). Perinuclear trafficking and subsequent pooling of these endosomes resulted in variability in AD by the internalized activity fraction (Fig. 7C), which was found to be time-dependent (Fig. 8). This indicates that the assumption in the geometric model that activity is uniformly distributed in the cytosol is an inaccurate model for antibody internalization. This finding is in agreement with previous suggestions [9].

The mean ADs for the main compartments are log-normally distributed, which is in agreement with previous observations [32–34].

We have found that the application of microdosimetry resulted in a significantly better prediction of SF, relative to using non-stochastic mean ADs (i.e., s-values). This can be explained with the highly non-linear behavior of microdosimetry, especially at lower activity concentrations. For example, the 0-hit likelihood is greater than 50% for the lower half of activity concentrations that were modeled (Fig. 11). In an average AD approach, this effect would be ignored, and the imparted energy would essentially be averaged over all cells, resulting in lower ADs per cell. Consequently, given a certain observed SF for that activity concentration, this would result in lower estimates for cell sensitivity to radiation. Therefore, we concluded that a microdosimetric approach is needed for the range of activity concentrations used in a typical cell survival assay.

The analyses in this study were based on the observation of 567 individual cells under experimental conditions. This allowed for a rigorous statistical approach, which included the ability to estimate 95%-confidence intervals for all of the calculated statistics, as well as formally test for significance between groups. As is evidenced by the narrow confidence intervals as well as statistically significant tests, the number of cells we have included for this study was sufficient to support the statistical claims made in this manuscript.

There are several simplifying assumptions that underpin this study. We have posited that cell kill is caused by DNA damage and that therefore the nucleus is the sole radiobiologically relevant target for αRPT. Although this is a common assumption, we acknowledge that there are many other radiobiological pathways which can lead to apoptosis. For example, ionizing radiation absorbed by the cell membrane can cause lipid rafts, mitochondrial damage can result in the release of cytochrome c, the bystander effect can result in the production of reactive oxygen species and there are many more [35]. However, to include these in a radiobiological response model, the quantitative alpha particle absorbed dose–response relationships for each of those pathways (and their interactions) would be needed. As far as we are aware, there are currently no such quantitative relationships and the (differential) activation of each of these pathways will need to be established in future work.

We have used the Ab locations as a proxy for the location of all isotope decay events. As such, the diffusion of daughter isotopes away from the original Ab location was assumed to be negligible, which might be reasonable given the modest half-life of the daughters of ^212^Pb. Furthermore, adding diffusion processes would require estimating currently unknown model parameters, such as the (likely anisotropic) diffusivity of the different daughter isotopes through a cell monolayer and the probability of daughters escaping the chelator through nuclear recoil. Some work has been done on the latter to increase retention of daughters [36]. We implicitly assumed that after internalization, the Ab-isotope construct remains either fully intact or when it dissociates through biological processes, the Ab, isotope and its daughters all stay within the endosome and are later pooled into lysosomes [37]. The fate of the isotopes after internalization remains unclear. The quantitative data which would be required for additional layers of model complexity is not available. Although this simplification might have some impact on the exact the AD estimates we present here (especially the relative contribution of the membrane source region), we believe the comparison to the geometric model as well as the main findings of this work are not significantly affected by this.

We further acknowledge that the binding and internalization kinetics used in this study for AD modeling are specific to Ab-mediated targeting and could be different for peptides or small molecules. However, as the underlying physics do not change, we expect that many of the observations (e.g., the importance of cell clustering behavior and microdosimetry) will generalize to other alpha-emitter radiopharmaceuticals.

## Conclusion

We have shown that cell clustering and stochastic radiation effects, rather than cellular geometry, are the main drivers of inaccuracies in current geometric models. Radiobiological response models at the cellular level should incorporate these effects when the aim is to generalize the findings to other experimental designs or when investigating radiobiology in vivo.

## Supplementary Information

**Additional file 1**:** Figure S1**. Additional method details.

**Additional file 2**: Timelapse of antibody binding and internalization.

## Data Availability

The datasets used and/or analysed during the current study are available from the corresponding author on reasonable request.

## Abbreviations

AD: Absorbed dose
RBE: Relative biological effectiveness
SF: Surviving fraction
αRPT: Alpha-emitter radiopharmaceutical therapy
EBRT: External beam radiotherapy
Ab: Antibody
95%-CI: 95% confidence interval
