# Supplementary material for: Large-scale in vitro microdosimetry via live cell microscopy imaging: implications for radiosensitivity and RBE evaluations in alpha-emitter radiopharmaceutical therapy
Source: J Transl Med. 2023 Feb 24;21:144. doi: 10.1186/s12967-023-03991-1 (PMC9951424; doi:10.1186/s12967-023-03991-1)
Supplement: Supplementary file 1 — Additional file 1: Figure S1. Additional method details. [file 12967_2023_3991_MOESM1_ESM.docx]

# Additional files

## Photobleaching correction


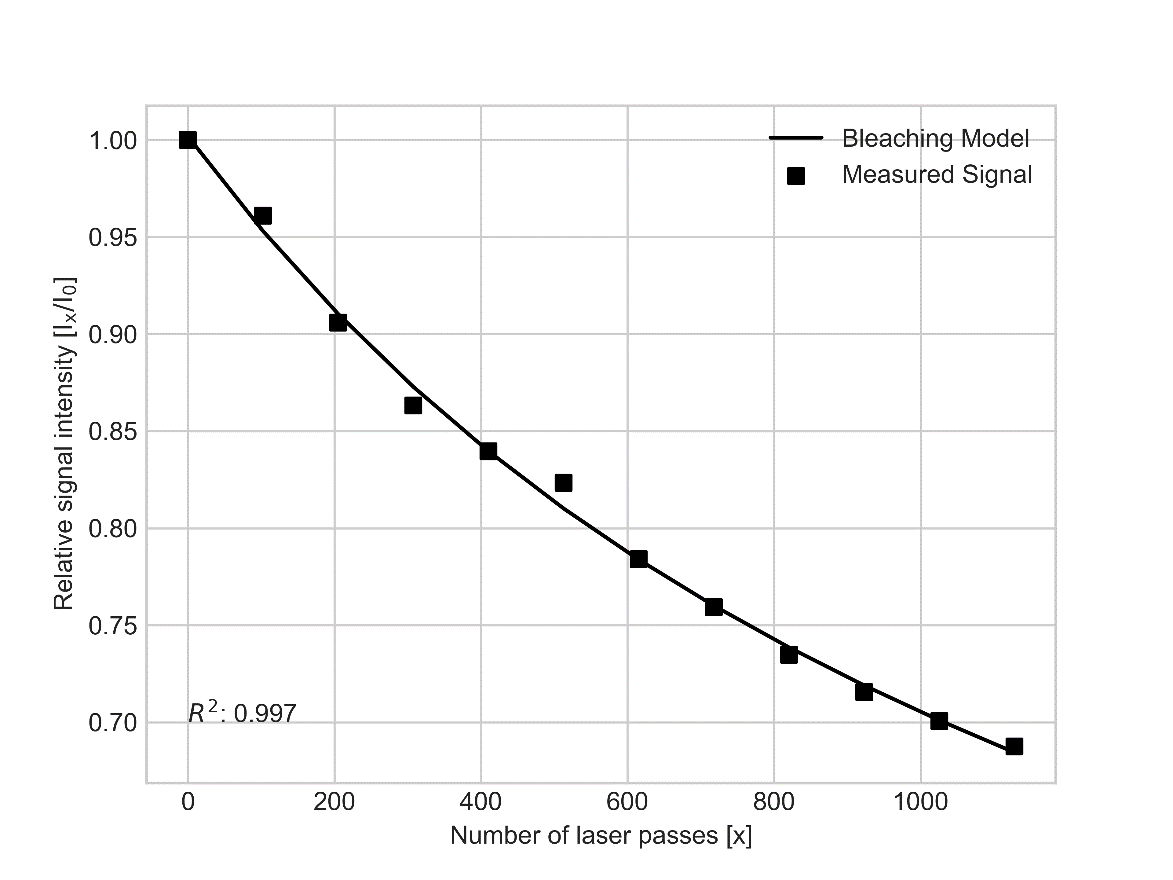


Figure S1 Photo bleaching characteristic curve.

$$\frac{I_{x}}{I_{0}}=\left( ae^{-bx}+ce^{-dx} \right)$$

With x the number of laser passes through a voxel, I_x_ the fluorescence signal intensity after x number of passes, and I_0_ the initial fluorescence signal intensity. Parameters found: a = 7.71x10^-1^, b = 1.51x10^-4^, c= 2.31x10^-1^, d=1.70x10^-3^.

## Cell segmentation

## Negative stain preprocessing

1. Normalize range (0-1)
2. Two passes of slice-by-slice CLAHE filter
3. Normalize range
4. Replace negative values with zeros
5. Add Laplacian
6. Gaussian blur
7. Add another Laplacian
8. Replace negative values with zero
9. Median filter with 3x3x3 voxel kernel

Cell geometry segmentation algorithm

Applied to preprocessed negative stain:

1. Otsu multiple thresholds 🡪 mask overlap with nucleus segmentation determines cell number
2. Watershedding to separate cells
3. Mauer distance transform
4. Watershedding again with (overlapping) nuclei as seeds
5. Binary opening (structure element: ball)
6. Connected component labelling, all masks smaller than 1E5 voxels were dismissed

## Nucleus segmentation

1. Rolling ball background removal
2. Otsu threshold
3. Filling voids
4. Signed Danielsson distance transform
5. Threshold distance map
6. Apply binary opening to thresholded map
7. Connected component analysis + reject extremely small components (noise)
8. Morphological watershed algorithm
9. Mask + connected components

This provided the initial nuclear masks. But due to differences in staining intensities, these needed the be refined further.

1. Center of mass of nuclear masks were presumed to be inside nuclei and acted as starting point for next steps
2. Initiate segmentation with small disk
3. Morphological Chen & Vese algorithm, 100 iterations
4. Filling voids

## Time frame coregistraton of nuclei

The following parameter settings were used in the SimpleElastix package for temporal coregistration of nuclei:

// *********************

// * ImageTypes

// *********************

(FixedInternalImagePixelType "short")

(FixedImageDimension 4)

(MovingInternalImagePixelType "short")

(MovingImageDimension 4)

// *********************

// * Components

// *********************

(Registration "MultiResolutionRegistration" )

(FixedImagePyramid "FixedSmoothingImagePyramid")

(MovingImagePyramid "MovingSmoothingImagePyramid")

(Interpolator "BSplineInterpolatorFloat")

(BSplineInterpolationOrder 1)

(Metric "VarianceOverLastDimensionMetric")

(Optimizer "AdaptiveStochasticGradientDescent")

(ResampleInterpolator "FinalLinearInterpolator")

(Resampler "DefaultResampler")

(Transform "BSplineTransform")

// Spline order of B-spline transform

(BSplineTransformSplineOrder 3)

// Enable/disable periodicity constraint of B-spline transformation

(UseCyclicTransform "false")

// *********************

// * Metric settings

// *********************

(MovingImageDerivativeScales 1.0 1.0 1.0 0.0)

(SampleLastDimensionRandomly "false")

(SubtractMean "true")

// *********************

// * Optimizer settings

// *********************

(NumberOfSamplesForExactGradient 2000)

(NumberOfResolutions 4)

(FinalGridSpacingInVoxels 10.0 10.0 5.0 1.0)

(GridSpacingSchedule 10.0 10.0 5.0 1.0 7.0 7.0 5.0 1.0 3.0 3.0 3.0 1.0 1.0 1.0 1.0 1.0)

(MaximumNumberOfIterations 500)

(SP_A 20.0)

(SigmoidInitialTime 0.0)

(AutomaticParameterEstimation "true")

// *********************

// * Mask settings

// *********************

(ErodeMask "false")

(ErodeFixedMask "false")

// *********************

// * Transform settings

// *********************

(HowToCombineTransforms "Compose" )

// *********************

// * Pyramid settings

// *********************

(FixedImagePyramidSchedule 8 8 1 1 4 4 1 1 2 2 1 1 1 1 1 1)

(MovingImagePyramidSchedule 8 8 1 1 4 4 1 1 2 2 1 1 1 1 1 1)

// *********************

// * Sampler parameters

// *********************

// The variance metric is allowed to get random samples, the penalty term isn't,

// because of the langrage multipliers, which should correspond to the same

// sample locations in every iteration

(ImageSampler "Random")

(NumberOfSpatialSamples 1000)

(CheckNumberOfSamples "true")

(NewSamplesEveryIteration "true")

(MaximumNumberOfSamplingAttempts 50)

// *********************

// * Output settings

// *********************

(WriteTransformParametersEachIteration "false" )

(WriteTransformParametersEachResolution "false" )

(WriteResultImage "false" )

(CompressResultImage "false" )

(WriteResultImageAfterEachResolution "false")

(ShowExactMetricValue "false" )

(DefaultPixelValue -1)
